# China is actively implementing health impact assessment legislation

**DOI:** 10.3389/fpubh.2025.1531208

**Published:** 2025-04-10

**Authors:** Quansheng Wang, Xiaoyi Zhang, Qi Zhang

**Affiliations:** ^1^Law School, Shandong University, Weihai, China; ^2^School of Physical Education, Shandong University, Weihai, China

**Keywords:** health impact assessment, legislation, primary health care and health promotion law, health, health equity in China

## Abstract

The health impact assessment system is imperative for prioritizing prevention and establishing “people’s health” as a central tenet. Implementing this system is contingent on the existence of “perfect legislation.” Although China has already begun to implement health impact assessment legislation, it is still in the exploratory stage and faces numerous internal and external legislative challenges. The civil and criminal legal norms supporting the health impact assessment must be established and perfected within the framework of developing the fundamental law. Supportive measures are being implemented, such as training skilled professionals, prioritizing underdeveloped regions, and using government evaluations and incentives, to promote the integration of health impact assessment into Chinese legislation. These efforts are directed at improving China’s legal system and health impact assessment coordination mechanisms, with the overarching objective of improving public welfare. This trend is in response to the growing social expectations of a better quality of life.

## 1 Introduction

In 2014, China implemented the “integrating health into all policies” strategy, which created opportunities to establish a health impact assessment (HIA) system. The outline of the “Healthy China 2030” plan issued in 2016 emphasizes the necessity of establishing a comprehensive HIA system (1). A systematic evaluation of the impact of various economic and social development plans, policies, and engineering projects on health is imperative. In 2021, the National Office of Health and Human Services and the Health China Action Promotion Office issued the “Notice on the development of the HIA system construction pilot work” and started the HIA pilot work.

Based on China’s current policies and regulations, the so-called health impact assessment (HIA) refers to public policies, plans and major construction projects that are issued or approved by the prescribed procedures, content, and methods, systematic investigation, analysis, and comprehensive evaluation of the potential health effects of the population and recommended activities.

After several years of exploration, the corresponding pilot work has been carried out to date in 32 mainland provinces in China (excluding Hong Kong, Macao, and Taiwan), and a certain amount of experience has been accumulated, making the time ripe for the establishment of a comprehensive legal system for HIA (2). In marked contrast to extensive and well-established practice, there are still some issues in the legislation of HIA in China, such as the need for greater standardization and operability. Compared to foreign countries, it lags (3). China’s legislation on the HIA needs urgent improvement.

This paper aims to systematically review the historical development of the Health Impact Assessment System, summarize the current legislative situation of the Health Impact Assessment in China and its legislative dilemma, and finally present the legislative ideas to improve the health impact assessment in China and the preliminary design of some important systems.

## 2 Development of a health impact assessment

In the 1980s, the HIA was a component of environmental impact assessment. It has been launched in developed countries such as Northern Europe and Australia. This parameter is used to assess the impact of large-scale infrastructure projects on health (4). Since the 1990s, with increasing concern about factors affecting health, HIA has developed rapidly. In 1993, the province of British Columbia, Canada, mandated that all bills submitted to the government by its Cabinet must be accompanied by an HIA report (5). The world’s first HIA tool was developed and released by the Ministry of Health and Elderly Management of Colombia (6). In 1999, the European Office of the World Health Organization issued the Common Protocol of Göteborg (7). The core values, the main procedures, and the primary HIA methods were proposed (8). In 2002, the EU incorporated the HIA into EU regulations. HIAs are explicitly required for legislation and sectoral policy development (9). In the Goldenberg Common Protocol, the World Health Organization (WHO) defines HIA as “evaluating the impact of a policy, plan, or project on a specific population.” A series of combined procedures, methods, and tools for assessing the potential impact on population health and its distribution in the population. In 2006, the International Association for Impact Assessment (IAIA) expanded the definition of HIA. HIA is a combination of processes, methods, and tools that can be used to systematically determine the potential (and often unintended) impacts on the health of a population. HIA can also determine the distribution of those impacts within the population. Finally, HIA can be used to identify responses to those impacts. HIA can be used when a policy is being formulated, a project is being implemented, or a plan is being prepared (10). The outline of the Healthy China 2030 Plan clearly states the need to establish a comprehensive HIA system. Systematically assess the impact of various economic and social development plans, policies, and engineering projects on health. The essence of the HIA system is to institutionalize health consideration throughout the formulation and implementation of public policy in various departments. It is essential to implement health policies and promote healthy city construction throughout the world. It is also a fundamental policy to implement the “prevention first” approach and advanced gateway development (11, 12). The international HIA system consists of two primary models. The first model is based on the breadth and complexity of health impact factors, highlighting the institutionalization of HIA and establishing a health impact assessment system independent of the environmental impact assessment. The second model integrates the engineering and project HIA into the environmental impact assessment system, without the establishment of an independent HIA system (13, 14). HIA in China is still in its infancy, and a comprehensive HIA system has not yet been established. Health impacts are addressed primarily through environmental impact assessments. Establishing a comprehensive HIA and evaluation system is fundamental for implementing the concept of “big health” and integrating health into all policies. The purpose is to eliminate all kinds of hidden danger that affect health at the source and maintain the “red line” of health.

The prevention of incidents that affect people’s health is a fundamental institutional arrangement (15, 16). Therefore, China must clarify its organizational system, determine the main body of promotion, specify the object and scope of the evaluation, and improve technical support to accelerate legislation on the HIA. Article 6 of the General Provisions of the Basic Medical and Health Promotion Law, which came into effect on July 1, clearly states that governments at all levels should establish an HIA system. This provision provides legal support to establish an HIA system in China. The same year, the State Council issued an opinion on deepening the Patriotic Health Campaign, calling for the establishment of an HIA system. We will promote a systematic assessment of the impact of various economic and social development plans, policies, and regulations, as well as significant engineering projects, on health. Efforts have been made to integrate health into all policies to enhance the clarity of the objectives and scope of HIA. In 2021, the State Council promulgated a notice on the pilot implementation of the HIA system. The overarching objective of this initiative is to establish a comprehensive and systematic HIA system throughout the country.

Since China introduced the strategy of building a healthy China, research on HIA in Chinese academia has been consistently deepened and expanded. This study provides solid theoretical support and information on the establishment and optimization of the HIA legal system. Chinese academics generally have a positive attitude towards the necessity of legislation on the HIA system. Institutionalization of the HIA field in China still needs to catch up with countries that developed it earlier. Considering the current realistic demands for the scientific and practical implementation of the HIA, it is particularly urgent to intervene and standardize construction at the legislative level. However, scholars have different views on the path to establishment, and these differences focus on the relationship between HIA and environmental impact assessment (17, 18). Some scholars argue that environmental and HIAs share similarities in terms of connections and objectives. Therefore, the content of HIA could be incorporated into environmental impact assessment, challenging the autonomy of HIA legislation (19, 20). Some scholars argue that the impact assessment of the health environment is extensive and that the influencing factors are not limited to the environment but also include social and economic factors. The sole environmental impact on health cannot encompass the entire scope of the HIA. Thus, HIA and current environmental impact assessment legislation must be separated (21, 22). Scholars advocate two legislative logics in this view; one being to enact a single HIA Act. One suggestion is to establish a dedicated section on HIA within current health legislation, such as the Law of the People’s Republic of China on Medical Health and Promotion of Health (23).

Little debate has been conducted on the content of this study, and most scholars agree that the health sector should lead the design of the HIA system. Attention must be paid to public participation, departmental cooperation, and information disclosure (24). In addition to this consensus, some differences in content and details will not be discussed in detail.

Most scholars studying HIA legislation are from public health, building science, and engineering. While only a few legal scholars have delved into this core content, at the end of the draft, these research findings are scarce and focus primarily on integrating health-related assessment mechanisms into the traditional environmental perspective. Separate legislation has not been established for HIA. However, other professional scholars have addressed the legislative perspective of HIA. It has yet to be the central focus of the article, and most scholars consider it a subsidiary component of the measures (25). Therefore, most of them are concise and summarized. From a legal perspective, this study focuses on developing a legislative framework for HIA, including specific laws. The proposed model has a significant reference value to legalize HIA and improve the HIA system.

## 3 Development of legislation and legislative efforts to assess the health impact in China

With the widespread adoption of HIAs worldwide, several countries and regions, such as Thailand, Canada, Britain, and Spain, have passed legislation to formalize HIAs. In 2007, Thailand included the relevant provisions of the HIA in its constitution. It is expressly stipulated that “any project or activity that significantly impacts environmental quality, natural resources, and community health shall also impact public health.” Impact assessment involves establishing the legal status of an HIA at the national level (26). In the same year, Thailand promulgated the National Health Act (27) to clarify the legal status of HIA. In 2002, Quebec passed the Quebec Public Health Act (28). Government departments and agencies should consult with the Health and Social Services Department regarding the sections of their acts or regulations that may significantly impact the population’s health. Comments from the Department of Social Services. In 2008, British Columbia, Canada, province amended the BC Public Health Act, stipulating that “all government departments should conduct HIAs on legislative projects that may affect public health” (29). In 2016, Wales introduced the Welsh Public Health Act (30). A statutory requirement has been proposed for public bodies to conduct comprehensive HIAs under specific circumstances. In 2011, Spain adopted the Spanish Public Health Act (31). It is proposed that all government departments conduct HIA on regulations, policies, plans, and projects that may have significant health implications. Andalusia, a province in Spain, has taken the lead in institutionalizing HIAs as required by law.

The China legal system for the HIA is still in the process of exploration. HIA directly impacts the lives and health of people in the jurisdiction. The application of this method usually includes various construction projects and urban planning (32). It is an important index and basis for evaluating the feasibility of construction projects and urban planning and for proposing improvement suggestions. Given this, it should be considered that the scope of the HIA falls within the legislative scope of cities, with districts and autonomous prefectures highlighted in the legislative framework, both in urban and rural areas. Construction and management, environmental protection, historical and cultural preservation, grassroots governance, and other related issues. At the same time, this study also retrieved and organized policy documents related to the HIA developed by municipal and higher authorities. The function of a control group enhances the understanding of current legislative data.

On 18 March 2024, this paper employed the law database of Beijing University, the database of Chinese national laws and regulations, and the national regulations database as its main research sources. Acquire legislation related to the HIA and relevant policy documents. With “health, ““health impact assessment” and “HIA” as keywords, an accurate search is conducted in the title and full text, respectively. The invalid, revoked, modified, and repeated search results are excluded. The search results exclude non-normative content, such as “letter, ““reply, ““notification, “and “case.” Finally, 23 pieces of legislation met the search criteria, including 7 laws (as shown in Table 1) and 16 regulations (see Table 2).

**Table 1:** Statistics of current laws related to HIA in China.

No	Name of the law	The subject of the formulation	Implementation time	Elements related to the HIA
1	Constitution of the People’s Republic of China (Revised in 2018)	The National People’s Congress	March 11, 2018	Article 33 of the Constitution refers to the State’s respect and guarantee of human rights. Article 21 states that the State develops medical and health services and protects people’s health, providing a fundamental legal basis for establishing an HIA system.
2	Civil Code of the People’s Republic of China	The National People’s Congress	January 1, 2021	Natural persons enjoy the right to health, which is protected by law, and no organization or individual may infringe on it. This provides the proper basis and legitimacy for the health impact on private law.
3	Basic Medical and Health Care and Health Promotion Law of the People’s Republic of China	NPC Standing Committee	June 1, 2020	Establish a principled norm for the health impact assessment system. However, there is no specific system.
4	Environmental Protection Law of the People’s Republic of China	NPC Standing Committee	January 1, 2015	To safeguard public health as the purpose of the law, the State establishes and improves its environmental and health monitoring system and encourages and organizes research on the impact of environmental quality on public health.
5	Law of the People’s Republic of China on the prevention and control of soil pollution	NPC Standing Committee	January 1, 2019	Legislation to protect public health The relevant departments will assess toxic and harmful substances in the soil. Based on its impact on public health and the ecological environment.
6	Law of the People’s Republic of China on the prevention and control of water pollution	NPC Standing Committee	January 1, 2018	A public health risk assessment and a management system for toxic and harmful water pollutants should be established to ensure legislative public health purposes.
7	Law of the People’s Republic of China on the Control of Atmospheric Pollution	NPC Standing Committee	October 26, 2018	A public health risk assessment and management system for toxic and harmful air pollutants should be established under legislation to protect public health.

**Table 2:** Statistics of current laws and regulations related to HIA in China.

No	Name of the statute or regulation	The subject of the formulation	Implementation time	Elements related to the HIA
1	Regulations on Henan Provinces’ Basic Medical Care and Health Promotion	Henan Provincial People’s Congress	June 1, 2023	Declarative provisions for establishing HIAs
2	Regulation of the Ningxia Hui Autonomous Region’s Patriotic Health Work	Standing Committee of the People’s Congress Ningxia Hui Autonomous Region	January 1, 2023	HIA of public policies and engineering projects
3	Regulations under the Patriotic Hygiene and Health of Shanghai Municipality Promotion	The Standing Committee Shanghai Municipal People’s Congress	November 1, 2023	HIAs shall be carried out for significant plans, policies and engineering projects.
4	Patriotic Health Regulations in Dongying City	Donging Municipal People’s Congress Standing Committee	March 1, 2024	Declaratory clause
5	Regulations on the Patriotic Hygiene of Jingdezhen Municipality	Standing Committee of Jingdezhen Municipal People’s Congress	December 7, 2022	Declaratory clause
6	Regulations in Linyi Municipalities in Healthy Villages	Linyi Municipal People’s Congress	July 1, 2021	Declaratory clause
7	Regulations on the Patriotic Health Work of Enshi Tujia and Miao Autonomous Prefecture	The Standing Committee of the People’s Congress of Enshi Tujia and Miao Autonomous Prefecture	December 8, 2023	HIA for significant policies, plans, and projects should be conducted.
8	Chizhou Patriotic Health Regulations	The Standing Committee of Chizhou Municipal Congress	November 1, 2023	Declaratory clause
9	Measures for the Patriotic Health of Sanya City (Revised in 2023)	The Standing Committee of Sanya Municipal Congress	September 21, 2023	Declaratory clause
10	Regulations on Patriotic Health Work in Nanchang City	The Standing Committee of Nanchang Municipal Congress	January 1, 2023	Establish an HIA system and build an expert database for relevant policies.
11	Nanning Patriotic Health Regulations	The Standing Committee of Nanning Municipal Congress	September 1, 2023	Conduct an HIA on Significant policies, Plans and projects.
12	Regulations on the Health of Shenzhen Special Economic Zone	The Standing Committee of Shenzhen Municipal Congress	January 1, 2021	HIA of planning, engineering projects, and normative documents involving public health shall only be promulgated or implemented if the assessment is qualified. The municipal government shall develop specific measures for HIA, and the municipal and district governments shall set up assessment expert committees to encourage social organizations and institutions to participate in the evaluation.
13	Measures by Zhejiang Province to Guarantee Rural Water Supply	The People’s Government of Zhejiang Province	February 1, 2024	Declaratory clause
14	Measures for the Administration of Administrative Normative Documents in Lianyungang City	Lianyungang Municipal Government	November 1, 2023	The HIA should be conducted to develop administrative normative documents on public health. The drafting unit shall provide materials for the HIA when submitting administrative normative documents Examination.
15	Administrative Measures for Patriotic Health Work in Longnan City	Longnan Municipal Government	November 10, 2023	Patriotic Health Committee is responsible for organizing HIA activities.
6	Measures for the Administration of Administrative Normative Documents in Jinchang City	Jinchang Municipal Government	August 18, 2022	The drafting department shall adopt an HIA to develop norms concerning public policies and significant engineering projects. When the drafting department submits its draft normative documents for examination, it shall provide materials for the HIA.

Although China’s focus on the HIA started relatively late, rapid development has been achieved due to the attention and promotion of the central government. The approach adopted by China is directed by the central government, with local governments and policies taking the lead. As early as 2007–2008, several documents on the HIA were initially issued. Among them, the outline of the second cycle of healthy urban construction in Jing’an District, described by the Shanghai District Government, took the lead in integrating health considerations into urban construction. The application of HIA With the promulgation of the “Healthy China 2030” plan outline and the accumulation of experience in pilot cities, legislation has gradually accelerated. In 2023 alone, 10 laws and regulations were promulgated explicitly mentioning the establishment of an HIA system. It is sufficient to demonstrate the importance attached to it. China has developed a set of fundamental laws, specifically the Law of the People’s Republic of China on Basic Medical Care and Promotion of Health (hereinafter referred to as the Health Law). The legislative framework for the HIA is beginning to take shape by implementing 20 sets of local laws and regulations (33).

## 4 The dilemma of HIA legislation in China

To shape the HIA legislative system with Chinese characteristics, HIA legislation in China needs to be improved with an adequate legal framework and complete content.

### 4.1 The paucity of HIA legislation is a concern and its classification is suboptimal, resulting in inadequate legalization

An issue is the scarcity of quantities. As indicated in Tables 1, 2, only one law on health promotion mentions the concept of HIA. The total number of laws and regulations is only 15, symbolizing that despite 31 provinces having conducted pilot work, most provinces and municipalities still need to translate the valuable experience into institutional safeguards for feedback on practice. However, all 16 pieces of legislation differ from a specific law on HIA. In contrast, 24 laws comprehensively address environmental impact assessment. There are thousands of laws and regulations, including one special law and six special regulations. The quantitative weaknesses of the HIA legislation were revealed through a comparison. The second issue is the low level of normative effectiveness. Within the legislation, the norms at the legal level are directly related to local laws and regulations. There should be more transition between administrative regulations and departmental rules. Most of China’s HIA norms require more legal validity. After searching, we found 510 policy documents on the HIA, accounting for 96% of all relevant texts. Taking into account the vast body of policy law, the perception of the existence of legislative norms is almost obliterated. Policy documents serve the unique purpose of improving the user experience when navigating a new system. They help mitigate risks and improve efficiency. However, policies may need to be developed to adapt to the diversity of interests under market conditions. This will hinder deep-rooted institutional innovation. Only when laws are abided by can there be good and effective governance. The most obvious consequence of the absence of laws is a weakened deterrence and a lack of binding force. Relying on temporary policies is a short-term solution (34).

### 4.2 The legal framework of China for the HIA must be more cohesive and complete

HIA is integrated as a core component of the concept of health under the Health Promotion Act. Health initiatives are prioritized and health principles are integrated into all strategies. This constitutes an expression of Article 6, with only 35 words in the entire text, which is simply a declaration norm. The same is true for other legislation. Throughout the health laws and regulation system, many sporadically include provisions stating that “an HIA system should be established.” The content of the system, the subject of evaluation, legal responsibility, and the steps to follow in the evaluation process are key aspects to consider. The answer cannot be found in the current legislation. The design of the system must address the cases encountered in HIAs that are documented and hindered by inaction. This weakens the legislative function and deviates from the original intention of “extending the law” and “being effective.” Refinement is an inevitable choice, but the process of evolution requires further discussion (35).

### 4.3 The local legislative HIA of China is rigid

Local government regulations have limited authority and supplement or elaborate on higher-level laws. In the absence of provisions of the superior law, more flexibility is needed, making it justifiable for legislation to align closely with the superior law. Consequently, the legislative initiative described in this section primarily targets local laws and regulations.

The number of 11 local laws and regulations is significant, but the level of innovation needs to be clarified. Although the legislative law emphasizes that local laws and regulations should generally not duplicate provisions already stipulated in superior laws, this principle must be implemented. Many only incorporate “forwarding” documents from administrative management into local legislation. As shown in Table 2, there is a high duplication rate between regulations and the overarching health laws. The provisions of the health law are broad enough to establish an HIA system. The government’s target responsibility assessment will include enhancing the primary health indicators of citizens. It is hoped that local laws and regulations will be tailored to the specific realities of each region. However, the local regulations of Chizhou, Dongying, Jingdezhen, and Henan province only emphasize the importance of conducting a “HIA.” The system for improving essential health indicators for citizens as part of the government’s accountability assessment must include significant detail. Among them, there is only a difference of two or three words between the original text of individual laws and regulations and almost identical health laws. The remaining local laws and regulations are also consistent across regions. This enhances the coordinating role of the evaluation object and the Aiwei Association, but lacks achievement with local characteristics. “Without local characteristics, local legislation will lose its value.” Liu et al. (36) state that “homogeneous decorative legislation not only weakens enforceability but also wastes legislative resources”.

Moreover, the content of local legislation on the HIA is conservative, and the main body of legislation consists of region-specific regulations. Therefore, local laws and regulations will be established differently. For example, the evaluation criteria for Shanghai, Enshi Tujia, Miao Autonomous Prefecture, and Nanning City include comprehensive planning, essential policies, and significant engineering projects. In Ningxia, evaluation focuses on public policies and major projects, while in Nanchang, only policies are evaluated. With the increasing interconnectedness of public affairs, which includes economic exchanges and environmental governance across various administrative jurisdictions, many construction projects, such as photovoltaic power plants and wastewater treatment facilities, often span multiple provinces and cities. How can you ensure compliance with local laws? There will be an embarrassed and contradictory between “different standards in the same case” and “different conclusions of colleagues”? Will the HIA incur high costs if conducted separately? In this way, traditional regulations of each government can only significantly decrease the impact of evaluation of specific projects (37).

### 4.4 The legislative framework for HIA in China needs to be improved

The CNKI database is a data source database. “HIA”, “health impact rating”, and “HIA” are search terms for precise subject searches. By eliminating laws, regulations, and documents that deviate from the subject headings, we obtained 176 documents in various forms. The retrieval date was March 30, 2024.

Theoretical research findings on HIA are relatively abundant. In addition to “HIA” and “health impact evaluation, “the top five keywords that frequently appeared in the literature were “urban planning,” “public health,” “healthy cities,” “health risk” and “environmental impact.” The evaluation, combined with the topics of the retrieved documents, reveals that the research focuses on assessing the health impacts and risks caused by individual projects such as urban planning, the environment, and urban construction. Methods and specific quantitative and qualitative aspects will be discussed in the 176 documents, with only 6.8% involved in in-depth research related to legislation or system construction. The findings reveal the need for research on HIA legislation. It cannot provide a strong legal foundation or technical support for legislation, which limits the legislative process to some extent.

### 4.5 Insufficient local legislative support for HIA in China

Local governments have responded to the central government’s call by conducting pilot projects and introducing implementation plans for the HIA system. However, the comprehensive support conditions at the grassroots level still need to be improved from a legislative perspective. This is a pain point that legislative work must consider.

The assessment link in HIA is at the core of the entire process. The expert committee typically performs this step to determine the outcome of the evaluation project. In some pilot cities, the expert evaluation process can be completed in less than a day. For various reasons, some experts lack a comprehensive understanding of evaluation policy. This makes the feedback results irrelevant, and the entire evaluation process becomes a formality (38).

The need for more talent is even more severe. HIA is not only related to basic fields such as public health and law; it also extends to urban planning, construction technology, environmental science, and other specific areas based on the characteristics of the evaluation object. The complexity of this demand indicates that the experts involved in the evaluation must have high levels of expertise. Few experts have this background; most are in universities and scientific research institutions in economically developed cities. It is not easy to find such talent in economically underdeveloped areas. Regional differences make it challenging to establish a comprehensive and uniform HIA system.

## 5 Development of HIA legislation in China

Only through legalization can the national governance system be standardized and refined. The HIA normative system remains dominated by policy and requires a corresponding legal framework, which hinders its ability to ensure long-term quality and effectiveness. It is easy to fall into the trap of governing as if in a perpetual campaign where legislation becomes the prevailing trend.

### 5.1 Formulating a specific foundational law for HIA

There are two types of HIA legislation in the world. One way to enhance the HIA is to recognize its independence. Another option is to integrate HIA into the environmental impact assessment (39). Although HIAs and environmental impact assessments do indeed have much in common, they were previously based on environmental impact assessments (40). However, this does not negate the essential difference between the two. International experience has shown that health considerations in environmental impact assessments typically focus on the effects of the physical environment, with limited attention given to the broader impact of the social and economic environment on health. The United States and Australia have integrated them and have gradually started independently practicing HIA (41). Furthermore, environmental impact assessment tends to be a positivist discipline, while social science disciplines influence HIA. The disconnect between the two fields indicates that environmental impact assessors should be more knowledgeable about the HIA. These are the inevitable drawbacks of integrating the two (42). According to the national conditions of China, the Environmental Impact Assessment (EIA) law excludes any provisions for HIA. This shows that, regardless of the cost of legislation, the corresponding content should be rewritten without concern for the cost of legislation. In conclusion, China should establish an independent HIA method.

### 5.2 Legislative mode: adopt a comprehensive legislative framework for laws

It is possible that the pilot process in China could have been executed with greater efficiency, thus exposing certain issues. Therefore, it cannot provide adequate empirical and theoretical legislative examples. In this case, it is imperative to respond at the legislative level and establish a foundation for implementing top-level design to force local governments to overcome bottlenecks. Therefore, a comprehensive legislative framework can be adopted to gather the commonalities of various HIAs during the initial legislative stage. As a fundamental and benchmarking standard, the exercise of dual functions is not only a component of the constitutional right to health and the declarative provisions of health law, but also an integral part of the constitutional right to health. It can also empower local practices and allow lower-level methods to adapt to changes in the situation. The increased inclusion of the law ensures its stability and respect for the authority of the rule of law.

Regarding structural arrangements, the framework of the environmental impact assessment law can serve as a reference. The concepts of health and the environment share similarities. For example, the right to health and a clean environment are fundamental human rights. The impact factors on health and the environment are complex. Implementing preventive measures in advance can lead to improved management and maintenance. Without legislation incorporating HIA as a primary component, HIA remains the only effective impact assessment method available. The legislative structure of the EIA law has a unique reference value (43).

Taking the EIA law as an example, the legislative structure of the HIA law is conceived as follows in the logical order of total points. The first chapter covers the general provisions. This includes legislative purposes and foundations, concept interpretation, evaluation criteria, fundamental principles, and advocacy standards. The second section discusses the HIA of the policy. The third section discusses the HIA of construction projects. The fourth section discusses the HIA of planning. The second and fourth sections cover the specific scope of the evaluation object, the evaluation content, the evaluation procedure, the relevant subjects, and their obligations. The fourth section discusses legal liability. The text covers the subject of responsibility, including its components and methods of assuming responsibility. The fifth section discusses supplementary provisions, such as authorization provisions, implementation timelines, and other ancillary matters that must be covered in the general and sub-provisions.

### 5.3 Main system design

#### 5.3.1 Basic principles

Legal principles serve as the foundation of the legal system, directly influencing its fundamental nature, core content, and value orientation. HIA falls under public health and should adhere to the legal principles outlined in health law. Furthermore, we should consider the unique values of risk prevention and the green economy represented by HIA and China’s focus on being ‘people-centered’. The development concept and strategic implementation of “Healthy China” are carefully chosen. According to the WHO, the core values of HIA are defined as “democracy,” “fairness,” and “sustainable development.” The principles of China’s HIA law should first be people-centered, health equity, health promotion, health priority, and prevention. Sustainable development, collaborative cooperation, comprehensive evaluation, and objective openness are essential (2).

#### 5.3.2 Appraisal object

According to international practices, HIA typically includes projects, plans, and policies. However, in the outline of the “Healthy China 2030” Plan, the evaluation objectives are more strictly defined. It is framed solely as economic and social development plans, policies, and major engineering projects (44). The evaluation objectives of local legislation and pilot projects vary within this scope. The 2030 Healthy China plan is a comprehensive long-term strategic health plan at the national level. This approach has a more forward-looking and practical significance in guiding the practice of HIA in China. Furthermore, the practice of HIA in China is still being explored and content settings should be carefully considered. Therefore, economic and social development plans, policies, and major engineering projects should be the primary focus of evaluation. The assessment is placed within the framework method and the specific scope of the assessment is subject to further refinement by the competent health department under the State Council in collaboration with relevant departments.

#### 5.3.3 Obligation subject and assessment procedure

**Figure 1:**
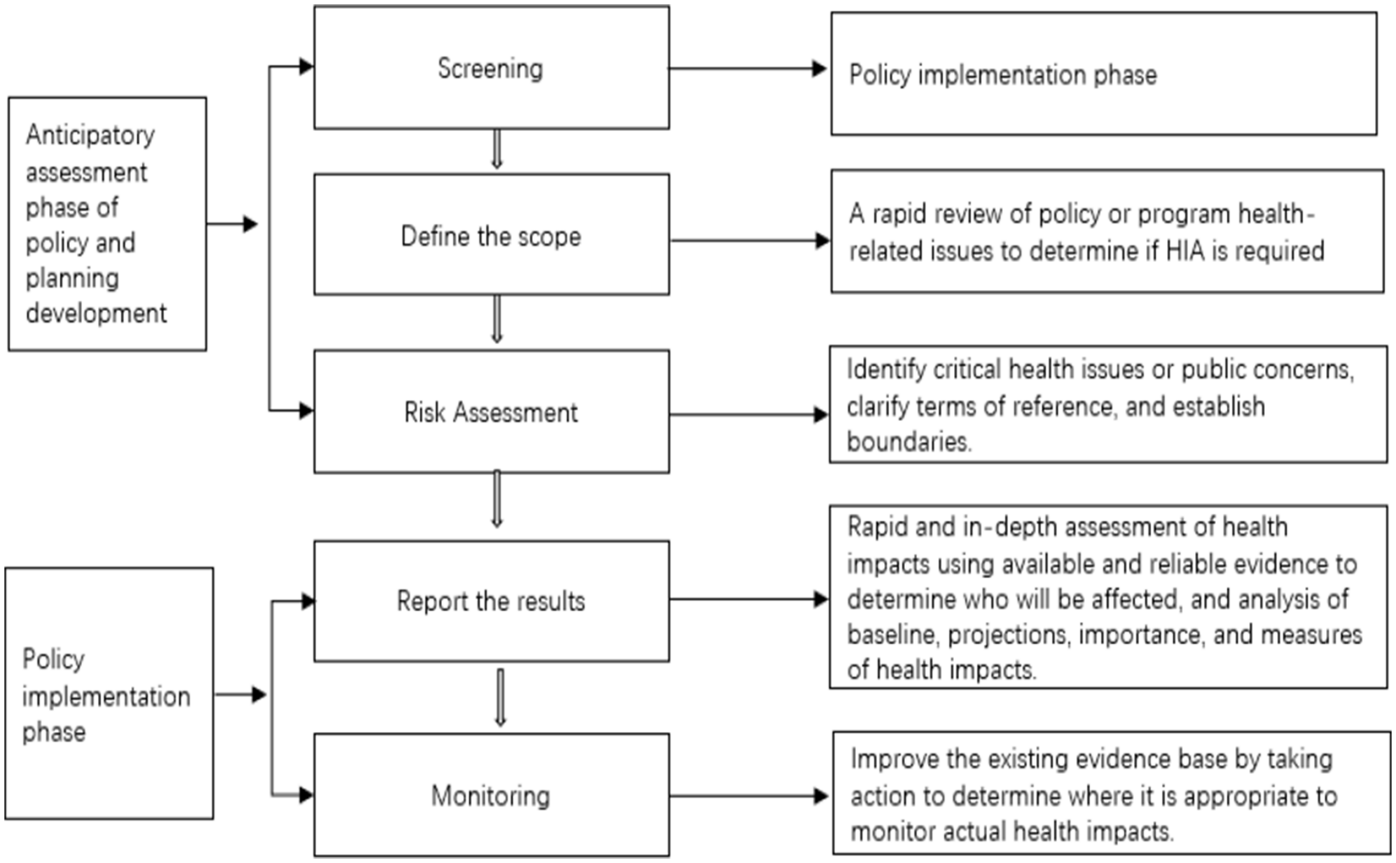
Basic procedures for assessing health impact.

A competent health department leads and coordinates the HIA and ensures quality control. The relevant departments responsible for developing policies or plans, in collaboration with professional assessment institutions, should conduct the HIA of policies and plans. The proportion of individuals evaluated by the organizations responsible for developing policies and plans should not exceed a specific threshold to maintain impartiality. The HIA report must be submitted to the planning policy and the examination and approval authority; otherwise, approval will not be granted. Regarding the HIA of a construction project, the construction unit delegates this responsibility to a professional assessment institution. Assessment institutions must have legal qualifications and should not be associated with organizations responsible for developing policy plans or with bodies involved in examination and approval procedures. The evaluation conclusions must be accepted. Before deciding, the examining and approving authority must organize an expert review while ensuring confidentiality. All HIA documents must be submitted to the higher health administrative department for records. Monitoring and evaluation will be conducted during the implementation of policies, plans, and construction projects to assess their impacts on health. Institutions and individuals will assume legal responsibility if they violate their legal obligations. The evaluation procedure should only address laws-relevant issues, such as the responsibilities of the departments mentioned above, the implementation process, and public participation (45). A more standardized HIA procedure was established, with detailed implementation steps and technical processes provided to the health department (see Figure 1). Establishing guidelines for action, technical specifications, and subordinate laws for HIA is more appropriate.

#### 5.3.4 Establish a system for public participation and information disclosure

All projects, plans, and policies directly related to public health rights and interests that are not confidential must be submitted for approval. Organizing hearings or other forms of active listening is necessary to gather public opinion on HIAs. Public opinions and the HIA documents should be submitted for approval, and the reasons for adoption or non-adoption should be explained. Establish a database of experts in counties (districts) to provide review support for HIAs before making approval decisions. Experts participating in the evaluation are selected from the expert pool based on their qualifications, and a specific percentage of external government experts are chosen randomly. The state will cover the payment of experts in a standardized manner. In addition to mandatory meetings, each department must organize demonstration meetings and symposiums periodically throughout the evaluation process. It is important to ensure effective safeguarding of the public’s right to participate and express themselves. The entire process must prioritize the participation of the public and improve information disclosure to increase public trust in the effectiveness of their participation. The examination and approval authority will publish the final evaluation results and establish a period for objections after publication to allow the public to exercise their oversight power. Public reporting and supervision channels should be established during follow-up planning, policy development, and construction project implementation (10).

#### 5.3.5 Establish a classification management system for HIA

Based on factors such as intensity, funding, and human resources, some countries categorize HIA into three types: rapid, intermediate, and comprehensive. There are similar methods in China’s pilot programs. The evaluation tools’ grades are integrated and selected based on the impact of policy changes on health, human resources support, and other variables. Using a focus on health priorities, classified management improves resource allocation efficiency. High-quality economic development and the concern for people’s health are combined with effective strategies. This practice can continue under the Basic Law. HIA is divided into two categories: comprehensive assessment, which involves a wide range of assessments, has strong comprehensiveness, and has the most extended duration, and key assessment, which focuses only on essential content. Rapid, short-time assessment (a simple and quick evaluation). Which form has finally been adopted? Health authorities can compare the dynamics of human, technical, and financial support, the impact on health, and the urgency of policy planning projects. The standard publishes a list of specific classifications (26).

#### 5.3.6 The relevant legal support system should be improved

The basic principles of the HIA cannot be fully explained if the supporting legislation lacks specificity and direction. No matter how detailed the basic law is, it can only serve as a solitary vessel and an “effective norm” on paper. It can only meet the specific requirements of some regions and policy-planning projects. We should fully utilize the universal applicability of administrative laws and regulations, the detailed “rehabilitation” function, and local laws and regulations. We should actively explore new approaches to collaborative legislation to ensure a comprehensive legislative system for HIA.

According to this assumption, the basic law is based on the framework of law. This implies that administrative regulations, second only to laws, are the first to perform the refinement task. The regulations on the HIA of policies, the regulations on the HIA of major construction projects, and social and economic development plans should be established. The regulations for the HIA outline the assessment steps and content tailored to the characteristics of each assessment object. It is convenient for all locations and disciplines to adhere to the implementation.

Local legislation on HIA should eliminate redundancy in the overarching legislation and address local issues. The existing norms of the overarching law have been adapted into local regulations that align with the economic and social development, production, lifestyle and national customs and habits of the city. In regions characterized by heavy industry, clearly defining the HIA of construction projects related to the environment is crucial. To enhance the adaptability and acceptability of central legislation in local areas and increase people’s willingness to abide by the law voluntarily.

The implementation of construction projects often crosses administrative boundaries. However, the fragmented legal system undermines the integrity of the HIA and hampers the effectiveness of a proper evaluation. The approval of “regional coordinated legislation” in the legislative law has introduced a new approach to solving this problem. Although there are still some disputes about the legitimacy and implementation of regional cooperative legislation, the first trial in Beijing-Tianjin-Hebei, the Yangtze River Delta, Guangdong Harbor, and other regions indicates that resolution of conflicts between local legislations, the establishment of an integrated regional legal environment, and the enhancement of regional in-depth cooperation is imminent (46).

#### 5.3.7 Coordinate with existing civil, administrative, and criminal law systems

The basic law on health impacts should emphasize the need to pay special attention to the right to health of specific groups, including children, women, the older adult, and disabled individuals. This will create a harmonious resonance with the protection of specific groups as outlined in the General Principles of Civil Law of China (27). The right to health in civil law theory is related to the preservation of physiological functions of citizens and the promotion of a sustained, stable, and positive psychological state. The Civil Code adopts this approach by stipulating that “the physical and mental health of natural persons shall be protected by law.” This aligns with the value orientation of the definition of health in the HIA. Establishing private rights and remedies for individuals involved in HIA can be facilitated by interpreting the right to health.

From an administrative law perspective, reviewing and approving the contents of an HIA are considered administrative acts. In particular, for an administrative license, the assessment and approval of the health impact of the project should adhere to the principles of the Administrative Licensing Law, which include fairness, transparency, and impartiality. In addition, the Administrative Procedure Law should define the “interested party” in the HIA with the plaintiff’s subject qualification. Residents can initially file a complaint with the HIA administrator. If the result of the complaint remains unsatisfactory, the complainant can take further action (18).

In criminal law, the provision of false HIA documents by personnel from professional assessment institutions can be considered a punishable offense—the types of activities included in the crime of providing false certification documents. Falsifying HIA documents directly related to people’s primary health rights and interests may be an aggravated offense (46).

### 5.4 Establishing implementation and enforcement mechanisms for China’s impact assessment legislation

#### 5.4.1 Clarify the regulatory body of China’s health impact assessment legislation and its scope of responsibility

Establish appropriate regulatory bodies for different assessment objects and the scope of assessment objects. If the health impact assessment involves planning and policies, it is the superior government department that formulates the plan or the government. The subject of supervision of construction projects is the central and local construction management departments at all levels. Health departments at all levels uniformly supervise the implementation of the health impact assessment system. The regulatory responsibilities of different regulatory departments should be clear.

#### 5.4.2 Strengthen the review of the evaluation report and publicize it to the public

The subject implementing the health impact assessment shall complete the health impact assessment report to a certain extent within a certain period of time and submit it to the corresponding health department. After carefully checking the health impact assessment, the health department shall disclose it to the public through the Internet, the media, and other means and accept the supervision of the society.

#### 5.4.3 Improve the accountability mechanism for evaluation

The accountability mechanism for the assessment includes not only the legal responsibility of the subject of the assessment, but also the legal responsibility of the implementation subject, such as the planning, policy-making, and review organs. The examination and approval authority, the construction authority, the supervisory authority of the construction project. The effective implementation of health impact assessment legislation can only be guaranteed by establishing a health legal accountability mechanism.

## 6 Conclusion

The most salient issue in China’s HIA legislation can be encapsulated as the absence of legal norms. The establishment of an autonomous EIA statute in 2002 signaled a pivotal shift in the nation’s legislative landscape. To further refine and strengthen this framework, it is imperative that China immediately enacts a foundational law on HIA, thus establishing a comprehensive and overarching legal framework for this crucial aspect of environmental governance. This fundamental legislative initiative will serve as the cornerstone on which the HIA system in China can be further developed and refined, paving the way for a more comprehensive and systematic approach to the protection of environmental health. The primary starting point of this document is to shape the legislative system of the HIA. However, because this paper only describes the general idea of the basic law of the HIA, some omissions could inevitably be made more explicit. It still needs to be followed up with a more rigorous and resilient portrayal of this unique system in the Basic Law. Protect the dignity of justice, well-being, and life.
